# High concentrations of cellulosic ethanol achieved by fed batch semi simultaneous saccharification and fermentation of waste-paper

**DOI:** 10.1016/j.biortech.2013.01.084

**Published:** 2013-04

**Authors:** Adam Elliston, Samuel R.A. Collins, David R. Wilson, Ian N. Roberts, Keith W. Waldron

**Affiliations:** The Biorefinery Centre, Institute of Food Research, Norwich Research Park, Colney, Norwich NR4 7UA, United Kingdom

**Keywords:** SSF, simultaneous saccharification and fermentation, SSSF, semi-simultaneous saccharification and fermentation, βG, beta-glucosidase (cellobiase), Simultaneous saccharification and fermentation (SSF), Cellulosic ethanol, Waste paper, Cellulase, Fermentation

## Abstract

A fundamental goal of second generation ethanol production is to increase the ethanol concentration to 10% (v/v) or more to optimise distillation costs. Semi simultaneous saccharification and fermentations (SSSF) were conducted at small pilot scale (5 L) utilising fed-batch additions of solid shredded copier paper substrate. Early addition of Accellerase® 1500 at 16 FPU/g substrate and 30 U/g β-glucosidase followed by substrate only batch addition allowed low final equivalent enzyme concentrations to be achieved (3.7 FPU/g substrate) whilst maintaining digestion. Batch addition resulted in a cumulative substrate concentration equivalent to 65% (w/v). This in turn resulted in the production of high concentrations of ethanol (11.6% v/v). The success of this strategy relied on the capacity of the bioreactor to perform high shear mixing as required. Further research into the timing and number of substrate additions could lead to further improvement in overall yields from the 65.5% attained.

## Highlights

► Batch addition of paper waste in SSSF results in up to 11.6% (v/v) ethanol. ► Low overall enzyme loadings (3.7 FPU/g substrate). ► High cumulative substrate loadings (65% w/v). ► High ethanol concentrations will improve distillation efficiencies.

## 1 Introduction

Most ethanol for transportation fuel is produced from starch or sucrose (first generation). These substrates can be employed in batch processes at relatively high concentrations facilitating high yields of ethanol at over 11% (v/v). This minimises the costs of distillation (Katzen et al., 2003). However, to enhance the sustainability of biofuel production, there is a desire to move away from crops relevant to human food, and there has been an international effort to enhance the efficiency of ethanol production from lignocellulosic waste streams from the agrifood chain (Waldron, 2010). Exploitation of such wastes has the potential to add value to food production and minimise the overall carbon footprint. Several demonstration plants have been recently constructed (Bacovsky and Worgetter, 2010). However, ethanol production from lignocellulose is not yet economically viable. There are a number of factors that make second generation approaches very expensive. These include the high cost of energy used in pretreatments, the difficulty of achieving sufficiently high substrate loadings, the cost and diversity of enzymes required for acceptable hydrolysis, the difficulty of effectively fermenting both hexose and pentose sugars, and the high energy costs associated with distillation of the low alcohol concentrations (Black and Veatch Limited, 2008).

The balance of these challenges is often waste-stream dependent. For example, waste paper and paper sludge from pulping do not require the energy-intense thermophysical pretreatments used to enhance enzymolysis of lignocellulose substrates. This is because they have already been “pretreated” by the pulping process which effectively de-lignifies the biomass and removes a significant amount of the poorly fermentable hemicellulose (Roberts, 1996). Very large quantities of waste paper and card are present in municipal waste streams. In the UK, for example, 12.3 M tonnes of paper waste was generated in 2008 (Defra, 2011), hence, a number of studies have been performed to evaluate the potential of ethanol production from these sources (Ballesteros et al., 2002; Chen et al., 2011; Dwiarti et al., 2012; Kang et al., 2011; Prasetyo et al., 2010). There have been continual improvements in the yield of ethanol from paper and sludge. Nevertheless, final ethanol concentrations achieved have been generally less than 1–2% by weight. This is mainly due to difficulties in achieving high substrate loadings. Above about 15% (w/v) the absorption of water by the paper results in a solid which requires very high forces for agitation and mixing as compared with the gelatinised starch or soluble sugar in first generation biorefineries. Furthermore, high lignocellulosic substrate concentrations are subject to the “solids effect” (Kristensen et al., 2009) in which expected glucose yields become reduced as substrate concentration is increased. Since paper waste contains cellulose at about 50% dry weight, a 15% (w/v) loading could not be expected to yield more than 3.75% (w/v) ethanol. One approach to addressing this problem involves the use of fed batch additions of substrate in combination with simultaneous saccharification and fermentation (SSF) or variations thereof. As saccharification proceeds, the cellulosic biomass is degraded. This will liberate more free water, reducing the viscosity or stiffness of the substrate suspension. The liquefaction could thereby facilitate further substrate addition, increasing the sugars available for fermentation. This was first demonstrated for paper wastes by Ballesteros et al. (2002) who achieved 1.8% (w/w) ethanol as did Kuhad et al. (2010). More recently however, Kang et al. (2011) achieved 7.6% (v/v) / 6% (w/v) ethanol from fed-batch SSF of paper mill sludges, although the process required an energy-intensive pre-de-ashing process. Nevertheless, the study demonstrated the potential to increase concentrations of ethanol derived from an insoluble cellulosic feedstock.

In the current study we have investigated approaches for fed-batch “saccharification and semi-simultaneous saccharification and fermentation” (SSSF) of shredded copier paper. The aim of the research has been to successfully achieve ethanol concentrations at levels comparable to those produced during first generation approaches whilst using minimal quantities of commercial cellulases. This provides a basis for reducing the costs of distillation (Hengstebeck, 1961; Katzen et al., 2003).

## 2 Methods

### 2.1 Materials

Commercially available cellulase Accellerase® 1500 (Genencor, Rochester, N.Y., USA); *Trichoderma reesei* and accessory enzyme β-glucosidase (βG) – Novozyme 188 (Novozyme Corp, Bagsvaerd, Denmark), were chosen for their high activities. These enzyme preparations were used “as provided” without any desalting or other purification steps. The substrate was M-Real Evolve Office 80 g/m^2^ paper (The Premier Group, Birmingham, UK); digestions and fermentations were carried out in 0.1 mol/L sodium acetate Buffer (Sigma Aldrich, Gillingham, UK).

### 2.2 Substrate preparation

M-Real Evolve paper was shredded using a PS-67Cs cross shredder (Fellowes, Doncaster, UK) to 3.9 × 50 mm particle size (Din Security Level 3), portioned into 125 g aliquots and sterilised by autoclaving in dry sealed bags (121 °C for 15 min).

### 2.3 Yeast preparation

Yeast (*Saccharomyces cerevisiae,* strain number NCYC 2826; National Collection of Yeast Cultures, Norwich, UK) was grown from a slope culture by inoculation into 1 L of Difco, Yeast and Mould (YM) broth (Fisher Scientific UK Ltd., Loughborough, UK): and allowed to grow over the period of ⩾3 days at 25 °C. The temperature was then reduced to 4 °C and the yeast was allowed to settle. YM media was decanted and the yeast cells reconstituted to 500 mL using yeast nitrogen base (Formedium, Hunstanton, UK). Prior to inoculation into hydrolysate the total viable count was measured using a NucleoCounter® YC-100™ (ChemoMetec, Denmark).

### 2.4 2 L reaction vessel

Initial studies were carried out using a 2 L fermenter (1.5 L working volume) equipped with a 502D agitator (LH Fermentation, Maidenhead, UK), an LH temperature regulator (LH Fermentation, Maidenhead, UK), a GFM17 mass flow meter (Aalborg®, US) and attached to an MX3 Bio sampler autosampler (New Brunswick Scientific, USA). Data were logged using Orchestrator software (Measurement Systems Ltd. (MSL), Newbury, UK). An additional condenser was installed in advance of the mass flow meter in order to prevent the expulsion of water vapour which would both decrease the sample volume and negatively affect the mass flow meter’s performance.

### 2.5 10 L reaction vessel

**Fig. 1:**
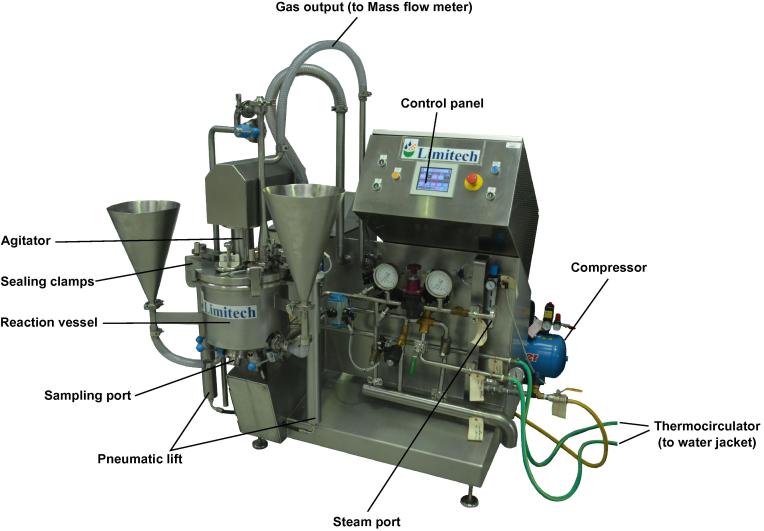
Tailored Limitech 10 L reaction vessel, with high torque stirring capability.

A tailored 10 L (5 L working volume) reaction vessel (Limitech A/S, Aabybro, Denmark) with additional computer control systems was used for additional study. It was equipped with a high speed mixer and a slow speed agitator (Fig. 1) and was temperature regulated using a Haake C35 (Thermo Scientific, Basingstoke, UK) circulator attached to a water jacket on the vessel. A GFM17 mass flow meter (Aalborg®, US) was attached to the gaseous vent at the top of the vessel and data logged using Orchestrator software (Measurement Systems Ltd. (MSL), Newbury, UK). Samples (10–15 mL) were taken during incubation from a tapped sampling point at the bottom of the vessel.

### 2.6 Initial vessel set-up

Shredded paper substrate was added to the vessel which was then brought to desired volume (1.5 or 5 L) with 0.1 mol/L NaOAc buffer (pH 5.0). The 2 L vessel was then autoclaved. This was not possible for the 10 L vessel which, instead, was heated to 90 °C for 10 min to sufficiently sterilise the initial buffer and paper substrate. The vessels were then equilibrated to 50 °C, the working temperature of Accellerase® 1500. Accellerase® 1500 (16 FPU/g of substrate) and βG (30 U/g of substrate) were added and stirred continuously.

### 2.7 HPLC – carbohydrate analysis

Samples (2 mL) were placed into sealed tubes and heated at 100 °C for 10 min to denature the enzymes and stop any further fermentation. Residual solids were then removed by centrifugation at 13,000 rpm for 5 min. Finally the supernatant was filtered using 0.2 μm syringe filters (Fisher Scientific UK Ltd., Loughborough, UK) into 300 μL glass vials (Essex Scientific Laboratory Supplies Ltd., Hadleigh, UK). Analyses of ethanol, glucose, xylose and cellobiose were carried out by HPLC using a Series 200 LC instrument (Perkin Elmer, Seer Green, UK) equipped with a refractive index detector. An Aminex HPX-87P carbohydrate analysis column (Bio-Rad Laboratories Ltd., Hemel Hempstead, UK) with matching guard columns was used, operating at 65 °C with ultrapure water as mobile phase at a flow rate of 0.6 mL/min.

### 2.8 GC – carbohydrate analysis

Solid residues were hydrolysed to monosaccharides using an adapted Saeman hydrolysis method (Saeman et al., 1945), 72% (w/w) H_2_SO_4_ at room temperature for 3 h followed by 1 mol/L H_2_SO_4_ at 100 °C for 2.5 h. These were then reduced with sodium borohydride (NaBH_4_) and acetylated by addition of 1-methylimidazole and acetic anhydride as described in Blakeney et al. (1983). The alditol acetates produced from the monosaccharides were then analysed by gas chromatography using a Perkin-Elmer Autosystem XL (Perkin Elmer, Seer Green, UK) and a RTX-225 (Restek, Bellefonte, USA) column.

## 3 Results and discussion

### 3.1 Paper composition

GC analysis revealed that M-Real copier paper had the following composition: 4.01% (w/w) moisture, 4.1% (w/w) Starch, 46% (w/w) cellulose, 11.86% (w/w) hemicellulose, 1% (w/w) Lignin and 33% (w/w) Kaolin/calcium carbonate, therefore a total glucan composition of 50.1% (w/w), comparable to other literature analyses (Wang et al., 2012).

### 3.2 2 L saccharification studies on batch addition to increase relative substrate concentration

Initial single time period saccharification studies (H1, H2 & H3, Table 1) were carried out using a 2 L vessel for 6, 12 and 120 h respectively. The levels of enzymes used were as recommended by the suppliers at 16 FPU/g cellulase, and 30 U/g βG. The suspended solids prior to digestion retained their structure with the paper fibres merely taking on liquid, after digestion however the consistency had achieved that of a viscous liquid. H1 and H2 both involved substrate concentrations at 5% (w/v) and resulted in final sugar concentrations of 7.5 mg/mL and 14.4 mg/mL respectively and yields equating to 30% and 57% (w/w) compared to a theoretical glucose maximum of 25.2 mg/mL. These initial experiments highlighted irregular and ineffective stirring; clumps of shredded paper substrate often became trapped within the vessel leading to unstirred areas. In an attempt to address this, digestions were carried out with a reduced substrate loading of 2.5% (w/v) which prevented clumping and thus enabled more vigorous and uniform stirring. The reduced substrate concentration rapidly resulted in a visually much more degraded sample (results not shown). On the basis of this the potential to increase the final glucose concentration by sequential batch digestions was considered. After 18 h digestion at 2.5% (v/v) substrate concentration, the undegraded solid material was removed by filtration through GF/C glass fibre filter paper. The supernatant was returned to the vessel along with an additional 2.5% (w/v) substrate, adjusted to 1.5 L volume with buffer and autoclaved. Once equilibrated to 50 °C further enzyme was added (Accellerase® 1500 16 FPU/g of substrate and βG 30 U/g of substrate) as before. This process was repeated to give a total of four additions resulting in a final glucose concentration of 30.8 mg/mL, equating to a yield of 61% (w/w) (H3, Table 1). This multiple addition method therefore increased both the effective substrate loading (10% w/v) and final yield of glucose (30.8 mg/mL).

### 3.3 Development of fermentation methodology

The final filtered hydrolysate from H3 was used to assess the potential for fermentation to ethanol using a high-ethanol-tolerant wine yeast. The supernatant was returned to the vessel and SHF performed by the addition of 200 mL of yeast inoculum (1.4 × 10^8^ cells/mL NCYC 2826 in nitrogen base). Over a fermentation period of 120 h, this resulted in an ethanol concentration of 1.2% (v/v) equating to 63% (v/v) yield from released glucose, 37% (v/v) yield from total glucose in the original substrate.

### 3.4 Fed batch saccharification followed by fed-batch simultaneous saccharification and fermentation in 2 L vessel (SSSF1)

**Fig. 2:**
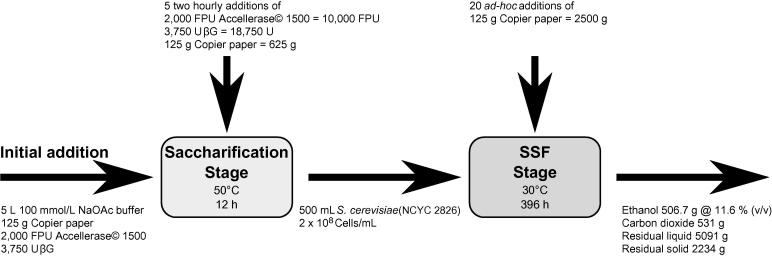
Flow diagram for SSSF with batch addition, input and output quantities are from SSSF 3.

**Fig. 3:**
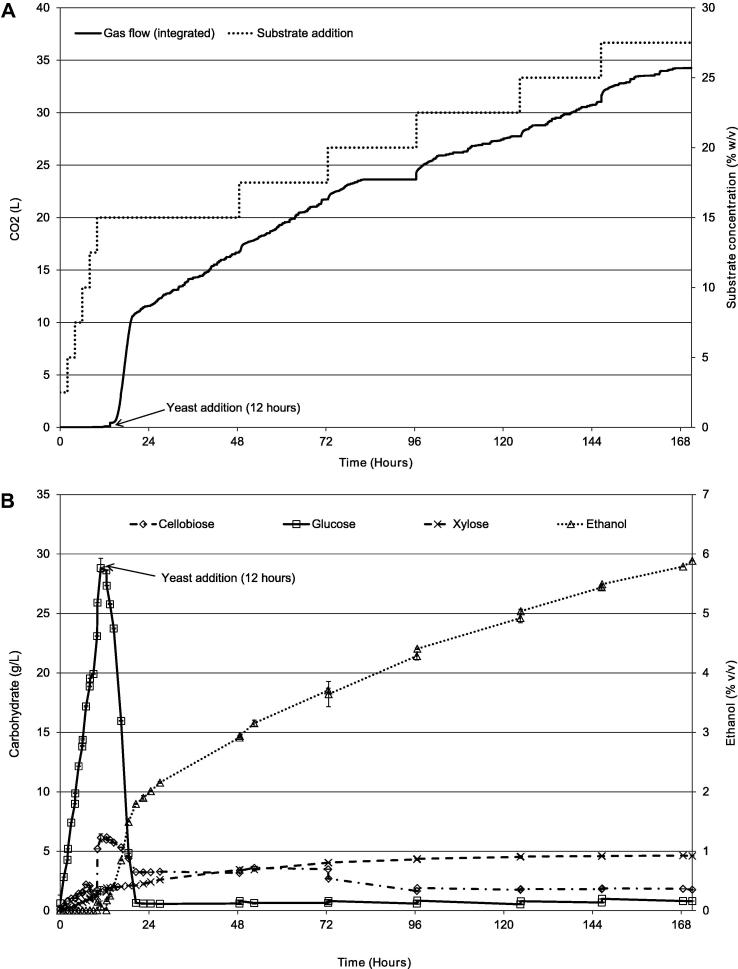
SSSF 1 (A) integrated gas output and substrate addition, (B) carbohydrate and ethanol production.

A fed-batch SSSF approach was conducted at the 2 L vessel scale (SSSF1, Table 1). In contrast to H3, undigested substrate was not separated from the soluble digestate. In order to promote yeast proliferation and fermentation, an initial hydrolysis phase was undertaken with sequential batch-additions of 2.5% (w/v) substrate, Accellerase® 1500 (16 FPU/g of substrate) and βG (30 U/g of substrate) at two hourly intervals at 30 °C. After 12 h by which time the glucose concentration had reached about 30 mg/mL (w/v), 200 mL of yeast inoculum was added (1.02 × 10^8^ cells/mL NCYC 2826 in nitrogen base). Further delay in initiating fermentation would run the risk of microbial contamination. Further substrate additions were made without additional enzyme. A flow chart describing the process is shown in Fig. 2. The timing of these additions along with ethanol, monosaccharides and CO_2_ production are shown in Fig. 3.

The results show that initial glucose accumulation permitted a rapid initial production of ethanol after which the increase in ethanol closely followed substrate additions. A final concentration of 5.9% (v/v) ethanol was achieved equating to a 65% (v/v) yield (maximum theoretical 8.96% v/v). The substrate addition was equivalent to final total of 27.5% (w/v) and the ethanol yield was similar to that achieved by Kang et al. (2011). After the eleventh addition stirring again became impaired by high viscosity which will have been due predominantly to undigestible kaolin and calcium carbonate from the paper substrate as well as any undigested cell wall material. At this stage, the digest presented a consistency similar to thick porridge, with little fibre degradation. Stirring moved the whole bulk and provided no counter flow. However the ethanol production had not plateaued at this time, suggesting that improved mixing might facilitate the further addition and digestion of substrate, and facilitate further ethanol production.

### 3.5 SSSF2 – scale up to higher shear 10 L vessel in order to increase workable substrate concentration

Due to the inability of the 2 L digester to mix the higher semi-digested solids loading, a specialised bioreactor with 10 L capacity (5 L working volume) was employed. This vessel, with its combined 550 W homogeniser/agitator and 4 kW scraped-surface paddle stirrers, was developed to enable the necessary mixing to be achieved and was based on heavy food processing equipment. This contrasts with the 2 L vessel which had been designed as a microbial bioreactor and therefore for stirring low viscosity cell cultures.

**Fig. 4:**
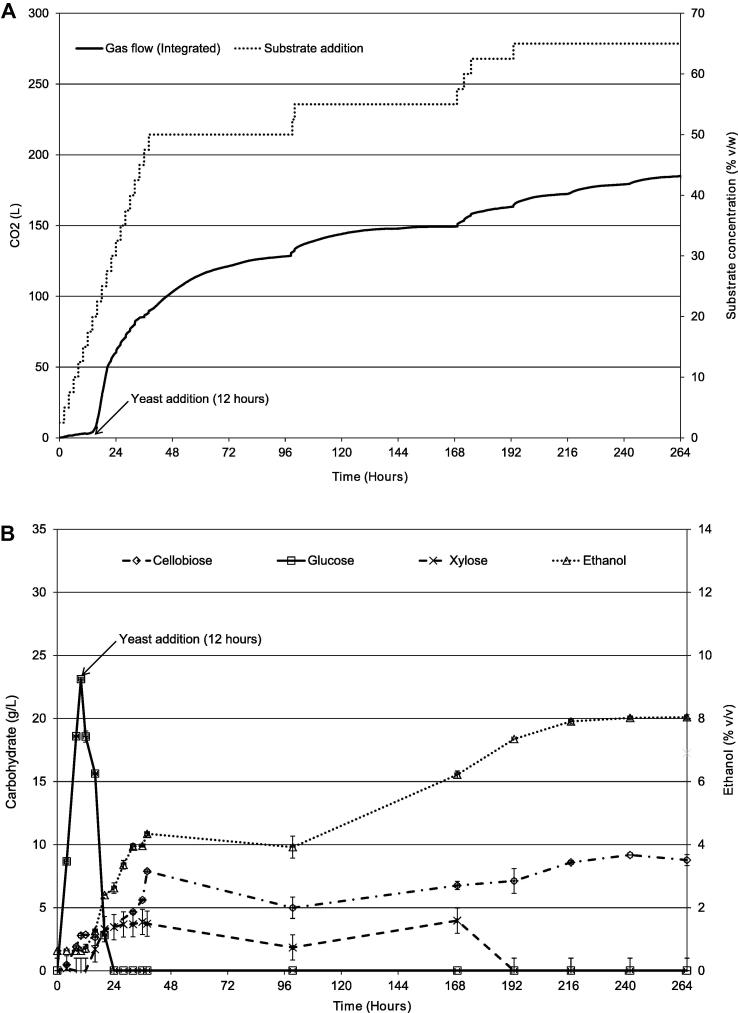
SSSF 2 (A) integrated gas output and substrate addition, (B) carbohydrate and ethanol production.

As for SSSF1, and as described in the flow diagram in Fig. 2, an initial hydrolysis stage was carried out to build up the glucose levels to initiate fermentation, although in this experiment, the digestion was carried out at 50 °C. The initial hydrolysis involved six two-hourly additions of 125 g (2.5% w/v) shredded copier paper substrate along with Accellerase® 1500 (16 FPU/g of additional substrate) and βG (30 U/g of additional substrate). These were added to 5 L 0.1 mol/L sodium acetate buffer, which enabled a total accumulation of 750 g substrate (containing 382.5 g cellulose; Fig 4) during the initial hydrolysis. Taking into account the hydration of the cellulose during hydrolysis, the theoretical maximum yield of glucose was 420.75 g glucose in 5 L total volume or 84.15 mg/mL. At the end of 12 h, a glucose concentration of 23.12 mg/mL was achieved (Fig. 4B, square symbols) equating to an initial yield of 27.5% (w/w).

The vessel temperature was reduced to 30 °C and 500 mL yeast inoculum (2 × 10^8^ viable cells/mL) was added which rapidly metabolised the available glucose (Fig. 4B). Subsequently the glucose concentration in the liquor remained low (less than 2.1 mg/mL), while ethanol concentration steadily increased. The subsequent saccharification of the substrate thus became the rate-determining factor in ethanol production, Substrate additions (125 g) were continued at 2 h intervals up to 40 h without any appreciable increase in glucose concentration. A total of 26 additions each of 125 g paper were made (20 in the fermentation stage and 6 during hydrolysis only) but no additional enzyme was added after the initial (pre-fermentation) hydrolysis stage. A final “accumulated” substrate concentration of ∼65% (w/v) was achieved in this experiment with additions totalling 3.25 kg. The concentration of ethanol estimated from carbon dioxide evolution was 9.5% (v/v) (Fig. 4A) compared to 8.0% (v/v) by HPLC (Fig. 4B). This difference was most likely due to the combination of both the marginal increase in volume due to addition of yeast, and the requirement of the vessel to be opened in order to add additional substrate, affecting the pressure of the system and also introducing some small quantities of oxygen to the system. The oxygen therefore allowing for standard respiration via the Krebs cycle, which although utilises less glucose, as explained by the Pasteur effect (Strathern et al., 1981), is likely to have also reduced the production of ethanol and thus the final concentration achieved.

The theoretical concentration of ethanol achievable with 100% (w/w) conversion to glucose and then onto ethanol can be calculated as in Eq. (1), where CP_s_ is the quantity of copier paper added to the system, in this case 3250 g, 50.4% (w/w) of which is cellulose 51.11% (w/w) of which can be converted into ethanol, 1.111 factor takes into account the water of hydrolysis (glucose, 180 g/mol/anhdryoglucose, 162 g/mol = 1.111), 930 g ethanol therefore being the theoretical maximum.

$$\text{Ethanol}\;(\text{g})=0.504\times {\text{CP}}_{s}\times 0.511\times 1.111$$

Equation 1. maximum theoretical ethanol.

The final volume of SSSF2 was 6700 mL, 28.53% of which was dry matter, and a liquid content of 5053 g with a volume of 4955 mL as determined from density measurements (density meter, Anton Paar DMA 5000, Anton Paar GmbH, Graz, Austria). This equates to a volume of ethanol of 342 mL or a mass of 270 g (based on 6.9% ethanol v/v), giving a final yield of 29%. It was also noted that the constant addition of paper every 2 h led to a highly viscous substrate after 20 additions, not unlike bread dough in consistency. This is likely to have retarded the enzyme digestion by reducing free movement and the availability of free water and possibly reducing the levels of free enzymes through non-specific binding.

### 3.6 SSSF3 – bespoke paper addition regime

SSSF2 showed that the addition of paper in a regimented two hour period eventually caused the substrate to become heavily thickened. Therefore a further regime was designed in which, subsequent to initial hydrolysis, phased additions were made at times where the material was deemed to have digested sufficiently. This was based on visual inspection of the mixture through a viewing port in the bioreactor.

**Fig. 5:**
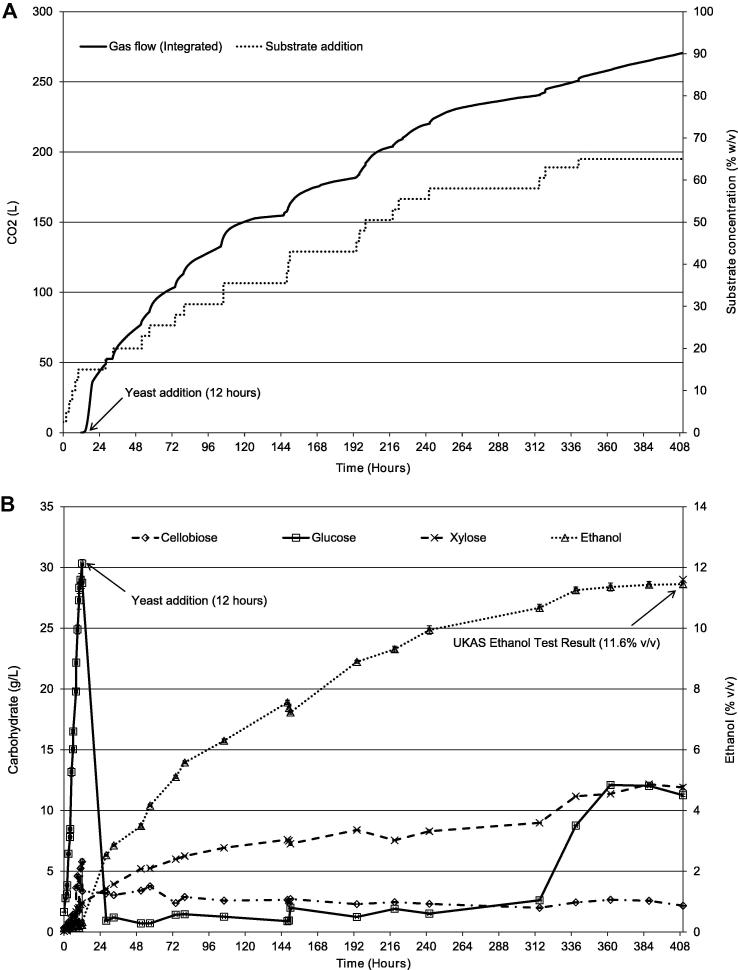
SSSF 3 (A) integrated gas output and substrate addition, (B) carbohydrate and ethanol production.

Following the approach described for SSSF 2 and in Fig. 2, an initial glucose concentration of 30.54 mg/mL was achieved in SSSF3 equating to an initial yield of 36.3%, comparable to that achieved in SSSF2. Again, the glucose concentration dropped sharply and remained low after the addition of yeast. However, addition of further substrate on a reasonably regular basis resulted in continual hydrolysis, fermentation, and production of ethanol (Fig. 5). Furthermore, after 315 h, the glucose level again started to rise, reaching 12.1 mg/mL by which time the final ethanol concentration was 11.6% (v/v) as quantified by HPLC (Fig. 5B). This was confirmed by Campden Technology Limited (Chipping Campden, UK) using their UKAS accredited TES-AC-567 method. The final ethanol yield was 54% (v/v of theoretical maximum). The increase in free glucose indicates that sufficient enzymatic activity remained within the reaction liquor and hydrolysis was not a limiting factor. Neither was the level of cellulosic substrate remaining (Table 2). Furthermore, the potential ethanol concentration estimated from carbon dioxide production, was 14% (v/v) (Fig. 5A) indicating that fermentation was sub-optimal. Hence, the suboptimal yield relates predominantly to yeast behaviour. It is possible that this was reduced due to ethanol-inhibition of the fermentation process. Alternatively, the long fermentation period may mean that the yeast had reached a steady state, and had lost vigour. The latter could well be the case if the supply of nutrients had fallen below critical levels. A higher efficiency of the order found in many other studies (Table 3) was achieved earlier in the fermentation, after 148 h and 14 additions, being 65.5% (v/v of theoretical maximum – based on a liquid content of 5.5 L).

The above results show that it is possible to achieve cellulosic ethanol concentrations of an order similar to that produced by first generation approaches by sequential batch addition of substrate with the use of robust agitation technology. The maximum concentration achieved in SSSF3 (11.6% v/v) was not optimal and there is room for further improvement by controlling substrate addition rates, initial enzyme concentrations and addition regimes, yeast strains (including high-temperature tolerant yeasts (Shahsavarani et al., 2013), and yeast nutrients. In addition, it is likely that different paper waste substrates will have an influence through the capacity of the insoluble kaolin/calcium carbonate to bind to the cellulose (Nikolov et al., 2000). Nevertheless, the ethanol concentration is very much higher than reported by other researchers working on paper or paper pulp waste streams (Table 3; (Ballesteros et al., 2002; Chen et al., 2011; Dubey et al., 2012; Dwiarti et al., 2012; Kang et al., 2010, 2011; Kuhad et al., 2010; Prasetyo et al., 2011; Sangkharak, 2011; Shen and Agblevor, 2011; Wang et al., 2012; Zhang and Lynd, 2010) highlighting the benefit of sequential batch addition and the crucial importance of effective agitation. Several other recent studies on lignocellulose waste streams have also focused on achieving higher ethanol concentrations. For example Lan et al. (2013) performed fed batch SSF of sulphuric acid-pretreated (180 °C) wood chips and achieved 47.4 g/L ethanol (6.0% v/v). However, the relatively high solids loading of 20% was achieved only after the vacuum rotary evaporation of sugar-containing acid pretreatment liquor. Prawitwong et al. (2012) exploited destarched, oil palm trunk alkali-pretreated tissues at 30% (w/v) substrate loading and high cellulase loadings (18 FPU/g) to create up to 8.5% (w/v) ethanol at yields of 68.8%. However, SSF was carried out in small reaction volumes of 70–80 mL in 100 mL serum bottles, shaken at 150 rpm. This would not be possible at industrial scale and a similar agitation through stirring would probably be challenging. Of course in the pilot plant approach reported in the present study involves considerable agitation. The energy that this consumes may be significant and will require further evaluation through life cycle analysis. However batch addition has allowed a “cumulative” substrate loading of about 65% (w/v) to be achieved which is considerably greater than previous reports (Table 3, (Modenbach and Nokes, 2012; Wang et al., 2012) and has been carried out at pilot-scale volumes. The approach has also facilitated relatively low enzyme usage. In SSSF3, 12,000 FPU cellulase (50 mL × 40 FPU/mL × 6 additions) and 22,500 U βG (15 mL × 250 U/mL × 6 additions) were added to the reaction resulting overall in 3.7 FPU/g substrate Cellulase and 6.92 U/g substrate βG, significantly lower than found in similar studies in the literature (Table 3). The low levels reflect the continued release of enzyme from the paper substrate as it is digested, and indicate non-permanent interactions with the inorganic components. The batch-addition regime utilised above appears to diminish the problems associated with enzyme blocking (Yu et al., 2012), where predominantly CBHs become non-productively bound to the substrate and therefore block attempts by other CBHs to productively bind to the substrate (Ma et al., 2008). The addition of new substrate increases the number of active sites in the mixture therefore allowing hydrolysis to continue despite blocked sites on the original substrate. The reduction of competition for relatively few active sites, by addition of new ones may also enable previously blocked enzyme to recommence hydrolysis and eventually detach from the substrate.

The time of the SSSF may be seen to be currently disadvantageous. SSSF3, for example, was performed for over 400 h. Nevertheless, the shape of the ethanol curve (Fig 5B) indicates that the bulk of the production might be achieved within less than half the time.

Finally, it was observed after experimentation that the recalcitrant material from SSSF was bright white in colour, suggesting that it was made up predominantly of calcium carbonate, as would be expected. This observation leads to the possible re-use of this by-product as a paint additive (Carr & Frederick, 2000) in addition to being re-used in the paper making process.

## 4 Conclusion

Batch addition of shredded copier paper in SSSF improves the final ethanol concentration. Early additions of enzymes followed by substrate only addition enables low overall enzyme loadings (3.7 FPU/g substrate) to be achieved. Stepwise substrate addition also permits high cumulative substrate loadings (65% w/v) by liquefying batches at each stage. This allows high levels of ethanol (11.6% v/v) to be achieved by increasing the solid substrate available for degradation. High ethanol concentrations will lead to improved distillation efficiencies though energy conservation. In order to achieve these results suitable equipment is needed to enable sufficient agitation at high substrate loadings.

## Figures and Tables

**Table 1:** Summary of hydrolysis experimentation – including enzyme and substrate loadings and final glucose and ethanol yields.

Ref.	Vessel (volume)	Enzyme concentration				Substrate			Glucose		Ethanol	
		Accellerase® (FPU/g)		βG (U/g)		Initial (% w/v)	No. of batch additions	Cumulative (% w/v)	mg/mL	Yield (% w/w)	% (v/v)	Yield (% v/v)
		Initial	Final	Initial	Final							
H1^a^	2 L (1.5 L)	16.0	16.0	30.0	30.0	5.0	1	5.0	7.5	30.0	–	–
H2^b^	2 L (1.5 L)	16.0	16.0	30.0	30.0	5.0	1	5.0	14.4	57.0	–	–
H3^c^	2 L (1.5 L)	16.0	16.0	30.0	30.0	2.5	4	10.0	30.8	61.0	1.2	37
SSSF1	2 L (1.5 L)	16.0	8.7	30.0	16.4	2.5	11	27.5	–	–	5.9	65
SSSF2	10 L (5 L)	16.0	3.7	30.0	6.9	2.5	26	65.0	23.1	27.5	6.9	29
SSSF3	10 L (5 L)	16.0	3.7	30.0	6.9	2.5	26	65.0	30.5	36.3	11.6	54

a 6 h.

b 12 h.

c 120 h (4 × 18 h).

**Table 2:** SSSF 3 sugar analysis of insoluble solids (HPLC), standard deviation in square brackets.

Time (h)	Carbohydrate (% w/w)							
	Glu		Xyl		Gal		Man	
1	66.60	[0.11]	12.78	[0.16]	2.97	[0.31]	6.33	[0.58]
12	56.51	[0.24]	9.80	[0.15]	1.28	[0.61]	4.65	[0.52]
28	49.80	[0.17]	8.82	[0.10]	1.64	[0.81]	5.38	[0.61]
194	43.06	[0.21]	7.27	[0.17]	0.04	[1.07]	4.55	[0.42]
315	46.86	[0.13]	6.87	[0.05]	<0.01	[1.01]	4.02	[0.26]
410	48.01	[0.22]	7.30	[0.09]	<0.01	[0.66]	4.43	[0.37]

**Table 3:** Summary of literature results, author’s results in bold for comparison.

References	Substrate		CellulaseFPU/g (min)	Glucose yield (% w/w) (max)	Ethanol	
	Type	(% w/v) (max)			Concentration	Yield (% v/v)
Ballesteros et al. (2002)	Paper sludge	10	15	47.9		79.7
Kang et al. (2010)	Paper sludge				45 g/L (5.70% v/v)	70.0
Kuhad et al. (2010)	Newspaper slurry	6	5	59.8	14.77 g/L (1.87% v/v)	
Zhang and Lynd (2010)	Paper sludge	17	10		40 g/L (5.07% v/v)	
Chen et al. (2011)	Pulped copier paper (de-ashed)	5	4–8	97.0		
Kang et al. (2011)	Paper sludge (de-ashed)	13.5	5		60 g/L (7.6% v/v)	70.0
Prasetyo et al. (2011)	Paper sludge		15		40 g/L (5.07% v/v)	66.3
Sangkharak (2011)	Waste paper		20	43.7	21.02 g/L (2.66% v/v)	43.7
Shen and Agblevor (2011)	Cotton gin/waste paper sludge	6	9.7		7 g/L (0.89% v/v)	78.5
Dubey et al. (2012)^a^	Waste paper (acid pre-treated)	12.5	n/a		3.73 g/L (0.47% v/v)	77.54
Dwiarti et al. (2012)	Paper sludge	5	15		11.34 kg/m^3^ (1.44% v/v)	80
Wang et al. (2012)	Waste paper (blended)	15 (High)	7.5	76.1		n/a
This paper	**SSSF 3 – Final**	**65**	**3.7**		**11.6% v/v**	**54.0**
	**SSSF 3 – Highest Yield**	**37.5**	**6.4**		**7.56% v/v**	**65.5**

a Hydrolysed by acid, not enzymatically.
